# Transcriptome profiling unveils the mechanism of phenylpropane biosynthesis in rhizome development of Caucasian clover

**DOI:** 10.1371/journal.pone.0254669

**Published:** 2021-07-13

**Authors:** Lingdong Meng, Xiaomeng Zhang, Lina Wang, Haoyue Liu, Yihang Zhao, Kun Yi, Guowen Cui, Xiujie Yin

**Affiliations:** College of Animal Science and Technology, Northeast Agricultural University, Harbin, China; Youngstown State University, UNITED STATES

## Abstract

Caucasian clover is the only perennial herb of the genus Leguminous clover with underground rhizomes. However, we know very little about its development process and mechanism. Transcriptome studies were conducted on the roots of Caucasian clover without a rhizome (NR) at the young seedling stage and the fully developed rhizome, including the root neck (R1), main root (R2), horizontal root (R3), and rhizome bud (R4), of the tissues in the mature phase. Compared with the rhizome in the mature phase, NR had 893 upregulated differentially expressed genes (DEGs), most of which were enriched in ‘phenylpropanoid biosynthesis’, ‘phenylalanine metabolism’, ‘DNA replication’ and ‘biosynthesis of amino acids’. A higher number of transcription factors (AP2/ERF, C2H2 and FAR1) were found in NR. There were highly expressed genes for R4, such as auxin response factor SAUR, galacturonosyltransferase (GAUT), and sucrose synthase (SUS). Phenylpropanoids are very important for the entire process of rhizome development. We drew a cluster heat map of genes related to the phenylpropanoid biosynthesis pathway, in which the largest number of genes belonged to COMT, and most of them were upregulated in R4.

## Introduction

Caucasian clover (Trifolium ambiguum Bieb.), also known as Kura clover) is the only long-lived leguminous clover with developed underground rhizomes and strong clonal reproduction ability. Its rhizome characteristics have been confirmed to be closely related to plant cold resistance [1], drought resistance [2] and grazing resistance [3]. Domestic and foreign scholars have conducted considerable research on the introduction [4], hybrid breeding [4–6], stress resistance [7–11], grazing utilization [12] and productivity [3, 13] of Caucasian clover. Due to the lack of genomic information, the molecular mechanism of rhizome formation in Caucasian clover is still poorly understood.

An increasing number of genes related to rhizome formation and development have been discovered and identified in different plants. Some genes are highly abundant in or specific to plant rhizomes, including energy and metabolism-related genes, such as monosaccharide transporter and methionine-S-methyltransferase in sorghum [14, 15] and β-glucosidase, starch branching enzyme and trehalose-6-phosphate synthase in bamboo [16]. In addition, plant rhizomes contain regulatory factors related to growth, such as elongation factors, tubulin and growth regulators in wild rice, and important transcription factors (TFs) are expressed in the root tip and elongation region of wild rice [17–22]. In addition, some resistance-related genes specifically expressed in rhizomes have also been identified in wild rice (Oryza longistaminata) [17–19], sorghum [20], lotus (Nelumbo nucifera) [21] and Phyllostachys edulis [22], such as peroxidase, L-ascorbate peroxidase, glutathione S-transferase, and catalase.

Phenylpropane biosynthesis is an important way to produce lignin. Lignin is a cell wall component, with cellulose and hemicellulose together constituting the main components of the plant skeleton. Lignin fills the cellulose skeleton, enhances the mechanical strength of plants, facilitates the transport of water in tissues, and resists adverse environmental conditions and invasion. It plays an important role in regulating cell morphology and development. As buds elongated, the expression levels of 16 lignin biosynthesis genes were upregulated in Bambusa, as determined by next-generation sequencing technology (RNA-seq) [23]. During the development of Raphanus sativus, a large amount of lignin accumulated in the leaves and roots. The lignin content increased significantly during the four stages of leaf development, while the accumulation of lignin decreased slightly in the thickening stage of roots [24]. To the best of our knowledge, there are relatively few studies on the complex lignin of rhizomes in Caucasian clover. It is possible to understand rhizome development by identifying key phenylpropanoid biosynthetic pathway genes.

Caucasian clover has no rhizome in the early stage but has only an upright main root and then grows a root neck, a horizontal root and rhizome buds in sequence. For a particular plant, the configuration of the root system can be stabilized until the mature stage. The root system of plants presents an axial root type at the young seedling stage, and it can produce obvious rhizomes until the mature phase [25]. Rhizomes appear in the mature phase of Caucasian clover. This study used RNA-seq technology combined with morphological and key gene verification analyses to study the different tissue parts of the roots (the mature phase root neck, main root, horizontal root, rhizome bud, and young seedling stage without the rhizome during the development of Caucasian clover). We identified differentially expressed genes (DEGs) in rhizome development, which served as the foundation for further exploration of the developmental mechanism of Caucasian clover and related gene function research.

## Materials and methods

### RNA sequencing and de novo assembly

**Fig 1:**
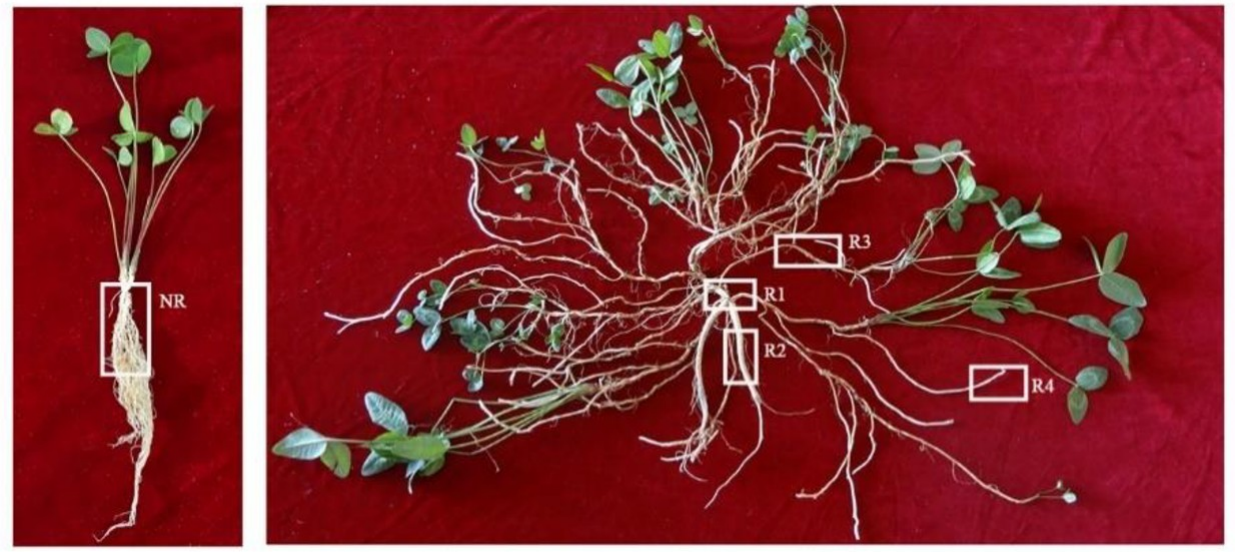
Schematic diagram of plant sampling.

Caucasian clover plants were collected from the wild cultivation nursery of Northeast Agricultural University (45°39′N, 126°30′E). The first sampling date was July 15, 2017. At the young seedling stage, the main roots of Caucasian clover were cut approximately 5 cm from the ground surface with a scalpel (NR). The second sampling was carried out on July 15, 2018. The root neck of Caucasian clover at the mature phase was cut with a scalpel (R1). In the mature phase, the main roots of Caucasian clover were approximately 5 cm above the surface (R2). In the mature phase, Caucasian clover grew horizontally from the main root, and the horizontal root was 5 cm away from the main root (R3). In the mature phase, buds approximately 1 cm from the horizontal root tips of Caucasian clover were formed (R4). Photographs of the specific locations of the five tissue sites sampled are shown in Fig 1. Plant materials were introduced from the Inner Mongolia Grass Variety Engineering Technology Research Center of Inner Mongolia Agricultural University, which performed formal identification of the samples, provided details of the specimens deposited and approved sample collection. The IPNI Life Sciences Identifier (LSID) for Caucasian clover is urn: lsid:ipni.org:names:522843–1.

Total RNA from each tissue was isolated using a MiPure Cell/Tissue miRNA Kit (Vazyme Biotech) following the manufacturer’s instructions. The Nanodrop, Qubit 2.0, and Agilent 2100 methods were used to detect the purity, concentration, and integrity of RNA samples to ensure that qualified samples were used for transcriptome sequencing. Complementary DNA (cDNA) library construction and normalization were performed according to published protocols. Three cDNA libraries (leaf, stem, and rhizome) were sequenced using an Illumina HiSeq X-ten platform, and paired-end reads were generated. Clean reads were obtained by removing adapter sequences, low-quality sequences, and sequences shorter than 35 bases. The remaining high-quality reads were assembled de novo into candidate unigenes using the Trinity programme.

### Determination of unigene expression levels

Because no reference genome was available for Caucasian clover, the clean reads from each sequencing library were mapped back to the assembled unigenes using Bowtie with a maximum mismatch of 2 nucleotides. The expression level of each unigene was normalized and calculated as the value of fragments per transcript kilobase per million fragments mapped (FPKM), which eliminated the influence of different gene lengths and sequencing discrepancies.

### Unigene annotation

The Basic Local Alignment Search Tool (BLAST) software was used to compare unigene sequences with the NR, Swiss-Prot [26], and KEGG databases [27]. KOBAS2.0 was used to obtain unigene KEGG orthology results in KEGG. After predicting the unigene amino acid sequence, HMMER software was used for comparison with the Pfam database to obtain unigene annotation information.

### Quantitative real-time PCR (qRT-PCR) validation of RNA-seq data

Ten DEGs involved in rhizome development were chosen for validation using quantitative real-time PCR (qRT-PCR). Primers for qRT-PCR were designed with Primer 3.0 software (http://biotools.umassmed.edu/bioapps/primer3_www.cgi) (S1 Table). qRT-PCR was performed using the ABI StepOne^TM^ Plus Real-Time PCR System with ChamQ Universal SYBR qRT-PCR Master Mix (Vazyme Biotech, Dalian, China), and the products were amplified with a mixture of 1 μL of cDNA template, 2× ChamQ Universal SYBR qRT-PCR Master Mix, and 0.4 μL of each primer (10 μmol/μL) in water to a final volume of 20 μL. The amplification program consisted of one cycle at 95°C for 30 s, followed by 40 cycles at 95°C for 10 s and at 60°C for 30 s. Fluorescent products were detected in the last step of each cycle. A melting curve analysis was performed at the end of 40 cycles to ensure proper amplification of target fragments. The melting curve analysis consisted of one cycle at 95°C for 15 s and then at 60°C for 30 s, followed by one cycle at 95°C for 15 s. qRT-PCRs for each gene were performed for three biological replicates, with three technical repeats per experiment. Relative gene expression was normalized by comparison with the expression of Caucasian clover (c257504.graph_c0) and analysed using the 2^−ΔΔ^CT method [28].

## Results

### RNA-seq statistical data

The total transcriptomes from the root neck (R1), main root (R2), horizontal root (R3), rhizome bud (R4) and rootless root (NR) were obtained using Illumina-based next-generation sequencing technology (RNA-seq) (Table 1). We obtained 25,396,794 reads from R1, 21,898,739 reads from R2, 24,268,708 reads from R3, 22,665,113 from R4, and 21,834,965 reads from NR. In total, we obtained 148.01 Gb of clean data with an average content of more than 41.51%, and each sample reached 6.09 Gb (a unit used to measure the amount of data, where 1 GB = 1,000,000 bp), and Q30 reached 90.48%. Clean data were assembled as described in the Methods section to generate 209,861 transcripts and 98,512 unigenes (assembled highly complex cDNA sequences) (S2 Table). Transcripts and unigenes had high assembly integrity, with N50 values of 1,854 and 1,598 and average lengths of 1158 and 846, respectively. The length distribution of the unigenes is shown in S1 Fig. The clean data of each sample were aligned with the assembled transcript or unigene library, and the results of the comparison are shown in S2 Table. Reads that are compared with transcripts or unigenes are called mapped reads, and mapped reads were used for subsequent analysis (S3 Table).

**Table 1:** Summary statistics of clean reads in the five tissues of the Caucasian clover rhizome.

	R1	R2	R3	R4	NR
Raw reads	25,396,794	21,898,739	24,268,708	22,665,113	21,834,965
Base number	7,599,474,508	6,546,248,531	7,263,680,664	6,785,234,564	6,534,299,629
Clean read	25,396,794	21,898,739	24,268,708	22,665,113	21,834,965
Clean read percentage (%)	99.39	99.22	99.38	99.26	99.22
Mapped reads	17,497,291	15,430,415	16,929,287	15,147,941	15,065,868
Mapped ratio (%)	68.90	70.46	66.76	66.83	69.00
GC percentage (%)	42.38	41.88	42.21	42.21	41.51
%≥Q30%	91.29	91.32	91.22	90.66	91.04

### Analysis of differentially expressed genes in different tissues

**Fig 2:**
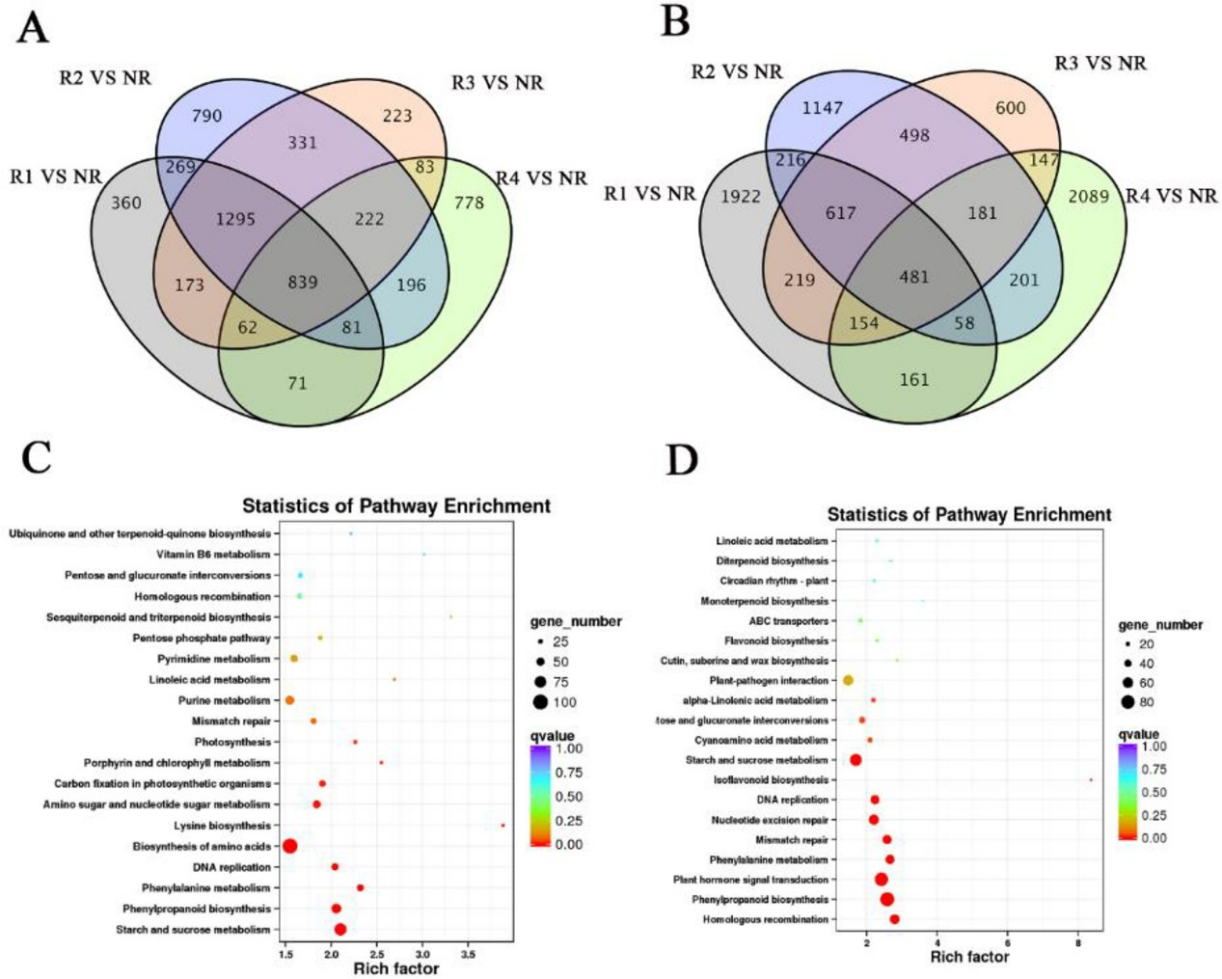
NR differential gene analysis. (A) Venn diagram of upregulated genes. (B) Venn diagram of downregulated genes. (C) KEGG pathway enrichment analysis of upregulated genes for NR. (D) KEGG pathway enrichment analysis of downregulated genes for NR.

We compared the up- and downregulated DEGs of NR tissues with those of other rhizome tissues (R1, R2, R3 and R4) (FDR<0.01-fold change>2) (Fig 2A and 2B). To study the DEGs in NR, we performed Kyoto Encyclopedia of Genes and Genomes (KEGG) pathway enrichment analysis of 839 co-upregulated (Fig 2C) and 481 co-downregulated genes in NR (Fig 2D). Most of the co-upregulated genes were enriched in ‘starch and sucrose metabolism’, ‘phenylpropanoid biosynthesis’, ‘phenylalanine metabolism’, ‘DNA replication’ and ‘biosynthesis of amino acids’. Compared with other rhizome tissues (R1, R2, R3 and R4), energy-related pathways were dominant in NR co-downregulated genes, mainly ’ homologous recombination’, ‘phenylpropanoid biosynthesis’, ‘plant hormone signal transduction’, ‘phenylalanine metabolism’ and ‘mismatch repair’.

For mature rhizomes, R4 had more DEGs than the other tissues (R1, R2 and R3), and the number of DEGs in R1 vs R4, R2 vs R4 and R3 vs R4 was 9,385, 8,884 and 7,352, respectively. R2 vs R3 had fewer DEGs (2,471; S4 Table).

**Fig 3:**
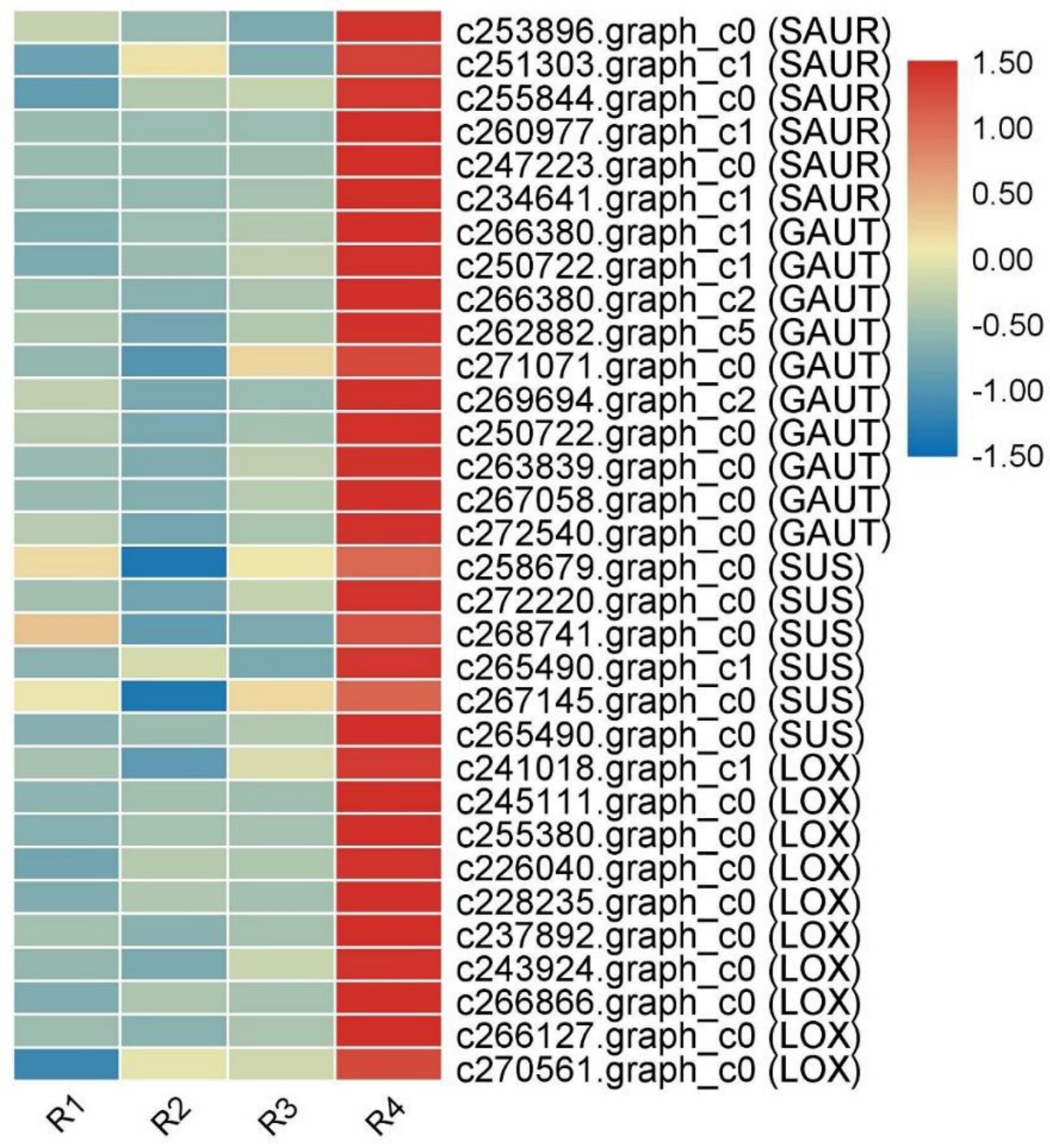
Heat map of highly expressed genes in R4.

We studied several genes that were more highly expressed in the buds (R4) and roots (R1, R2 and R3) (Fig 3). Most genes related to the starch and sucrose metabolism pathways, including the auxin response factor SAUR, galacturonosyl transferase (GAUT) and sucrose synthase (SUS), were upregulated in R4.

It is worth noting that lipoxygenase (LOX) genes, which are involved in linoleic acid metabolism and play an important role in the response to coercion, were also upregulated in R4. These genes are related to the formation of the rhizome buds of Caucasian clover.

The DEGs in the Caucasian clover rhizome (R1, R2 and R3) are involved in many molecular functions and metabolic pathways; many of these DEGs are transcription factors, such as bZIp, MYB and HD-zip, which are related to plant development and hormones and have been identified in previous studies on plant roots.

**Fig 4:**
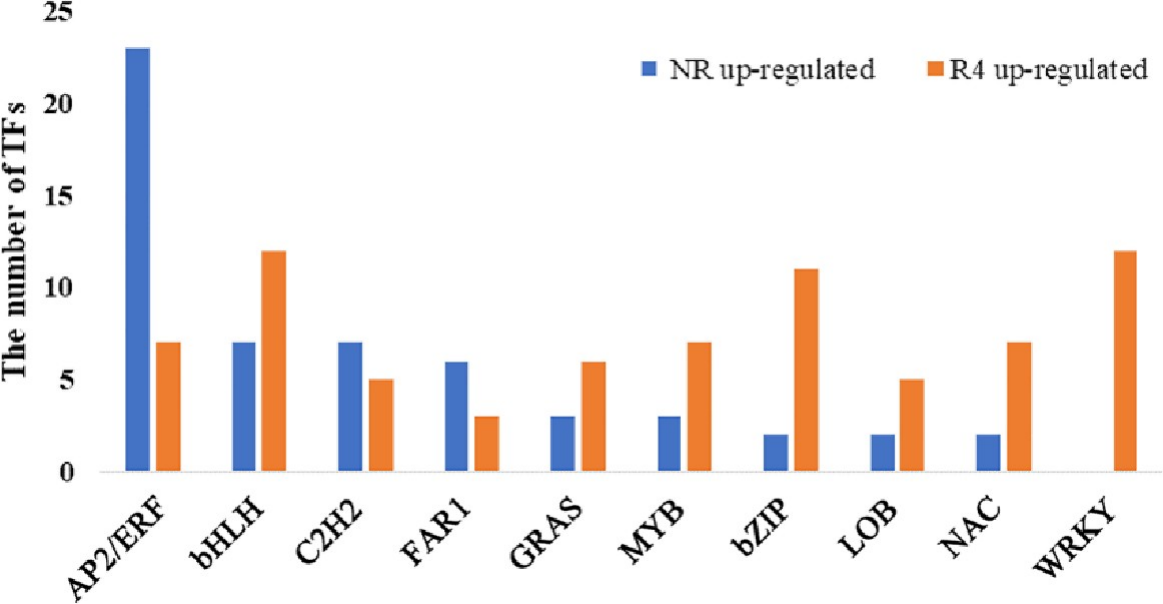
Bar plot of the number of transcription factors differentially expressed in R4 and NR.

Additionally, the DEGs in the two tissues (R4 and NR) that were transcription factors that regulate growth and development were significantly different (Fig 4). More AP2/ERF, C2H2 and FAR1 DEGs were found in NR, whereas more bHLH, WRKY and Bzip DEGs were found in R4.

### Differentially expressed genes in the five different tissues

**Fig 5:**
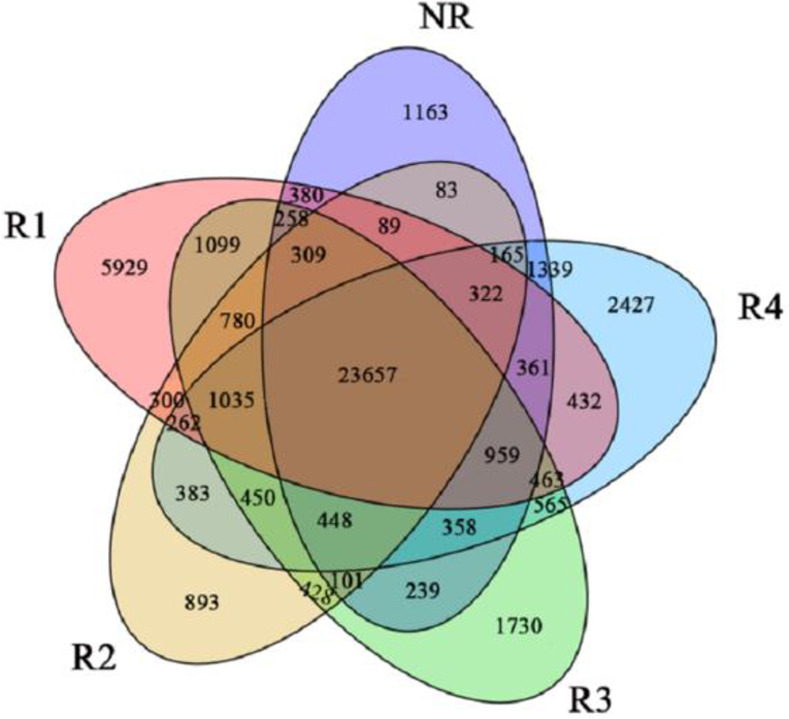
Venn diagram of gene expression in the five tissues.

We used a Venn diagram to show the number of DEGs identified for each part (each repeat of each tissue FPKM>0.1) (Fig 5). The FPKM (fragments per kilobase of transcript per million mapped reads) value was used to indicate the expression abundance of the corresponding unigenes. R1 and R4 had more genes uniquely expressed in those tissues (5,929 and 2,427, respectively), and the number of genes co-expressed in the five tissues was 23,657. Co-expressed genes may affect the overall development of Caucasian clover.

We selected some genes in different tissues that had FPKM values more than twice that of other tissues and may be related to rhizome development (Table 2). In R1, c273329.graph_c0 (ubiquitin-conjugating enzyme, UBE2D) and c250145.graph_c0 (RAC1, Ras-related C3) are related to the mitogen-activated protein kinase (MAPK) signalling pathway, which is a crucial pathway for plant root nodules and swelling.

**Table 2:** Statistical data of genes with higher expression in each tissue.

ID	R1	R2	R3	R4	NR	Gene description
c212626.graph_c0	727.57	0.17	17.89	0	0	-
c273329.graph_c0	34.07	0.00	0.54	0.11	0.05	UBE2D (ubiquitin-conjugating enzyme)
c250145.graph_c0	12.52	0.08	1.60	0.24	3.65	RAC1 (Ras-related C3 botulinum toxin substrate1)
c231546.graph_c0	23.30	0.07	0.83	0.53	0.39	THOC4 (THO complex subunit 4)
c244144.graph_c0	15.34	0.01	0.03	0.00	0.00	malZ (alpha-glucosidase)
c244261.graph_c1	125.87	307.42	138.12	126.78	115.26	KAR1 (ketol-acid reductoisomerase)
c247601.graph_c0	0.12	13.38	0.40	0.87	0.71	GST (glutathione S-transferase)
c270772.graph_c1	29.62	76.55	31.75	29.18	19.92	FBL (rRNA 2’-O-methyltransferase fibrillarin)
c270969.graph_c3	46.35	168.07	74.57	88.75	30.20	peroxidase
c212382.graph_c0	0.03	37.28	0.16	9.16	0.45	RFA1(replication factor A1)
c271019.graph_c0	23.46	39.54	100.17	32.66	21.80	CYFIP (cytoplasmic FMR1 interacting protein)
c266956.graph_c0	1.72	0.09	4.99	0.00	0.26	POR (NADPH-ferrihemoprotein reductase)
c158369.graph_c0	0.00	0.00	2.83	0.00	1.22	RLK-Pelle_RLCK-VIIa-2
c271751.graph_c1	4.22	1.94	22.21	4.62	3.53	RFA1 (replication factor A1)
c249389.graph_c0	0.01	0.02	4.40	1.10	0.58	fabF (3-oxoacyl-[acyl-carrier-protein] synthase II)
c260938.graph_c0	8.81	31.38	38.57	933.74	86.97	peroxidase
c268035.graph_c0	149.10	191.79	201.32	622.01	274.40	fructokinase
c260214.graph_c1	42.93	128.84	66.87	587.34	25.07	Xyloglucan (xyloglucosyl transferase)
c252926.graph_c0	11.74	6.05	19.17	393.08	18.82	peroxidase
c246619.graph_c0	13.60	32.17	36.37	383.67	28.41	peroxidase
c269011.graph_c0	13.68	12.65	11.08	300.55	7.44	peroxidase
c266443.graph_c1	12.29	1.39	4.55	294.37	4.83	peroxidase
c270344.graph_c1	1.97	1.02	3.39	141.15	6.77	IAA (auxin-responsive protein)
c271721.graph_c0	97.33	26.91	87.19	253.36	7.96	IAA (auxin-responsive protein)
c271111.graph_c0	39.29	11.07	63.75	242.37	19.63	PAL (phenylalanine ammonia-lyase)
c269539.graph_c0	216.73	248.56	207.66	101.39	2740.09	glgC (glucose-1-phosphate adenylyltransferase)
c261263.graph_c0	511.11	687.00	550.31	263.98	2000.90	GBE1 (1,4-alpha-glucan branching enzyme)
c260389.graph_c0	152.51	123.05	121.15	126.58	1123.70	glgC (glucose-1-phosphate adenylyltransferase)
c250773.graph_c0	7.94	17.81	7.69	31.72	189.47	MYB

In R2, some metabolic pathways are worth studying. Some key enzymes stand out, such as c244261.graph_c1 (KAR1) and c247601.graph_c0 (glutathione S-transferase, GST).

For R3, the number of DEGs related to molecular function was relatively high; these included c263901.graph_c1 (PPNA) and c158369.graph_c0 (RLK-Pelle_RLCK-VIIa-2).

Most genes with higher expression in R4 than in other tissues were related to peroxidase, xyloglucan and phenylalanine ammonia-lyase, which are closely related to stress resistance and some metabolic energy pathways.

Most DEGs highly expressed in NR are related to glycogen synthesis and metabolism. c261263.graph_c0 (1,4-alpha-glucan branching enzyme, GBE) is a key enzyme that catalyses glycosidic linkages of glycogen branches and is of great significance for biological energy storage.

c250773.graph_c0 (MYB) is a transcription factor and an important gene regulating rhizome growth and development. c269539.graph_c0 and c260389.graph_c0glgC (glucose-1-phosphate adenylyltransferase, glgc) are also a special class of genes that are highly expressed in NR.

### Analysis of the phenylpropanoid biosynthesis pathway

**Fig 6:**
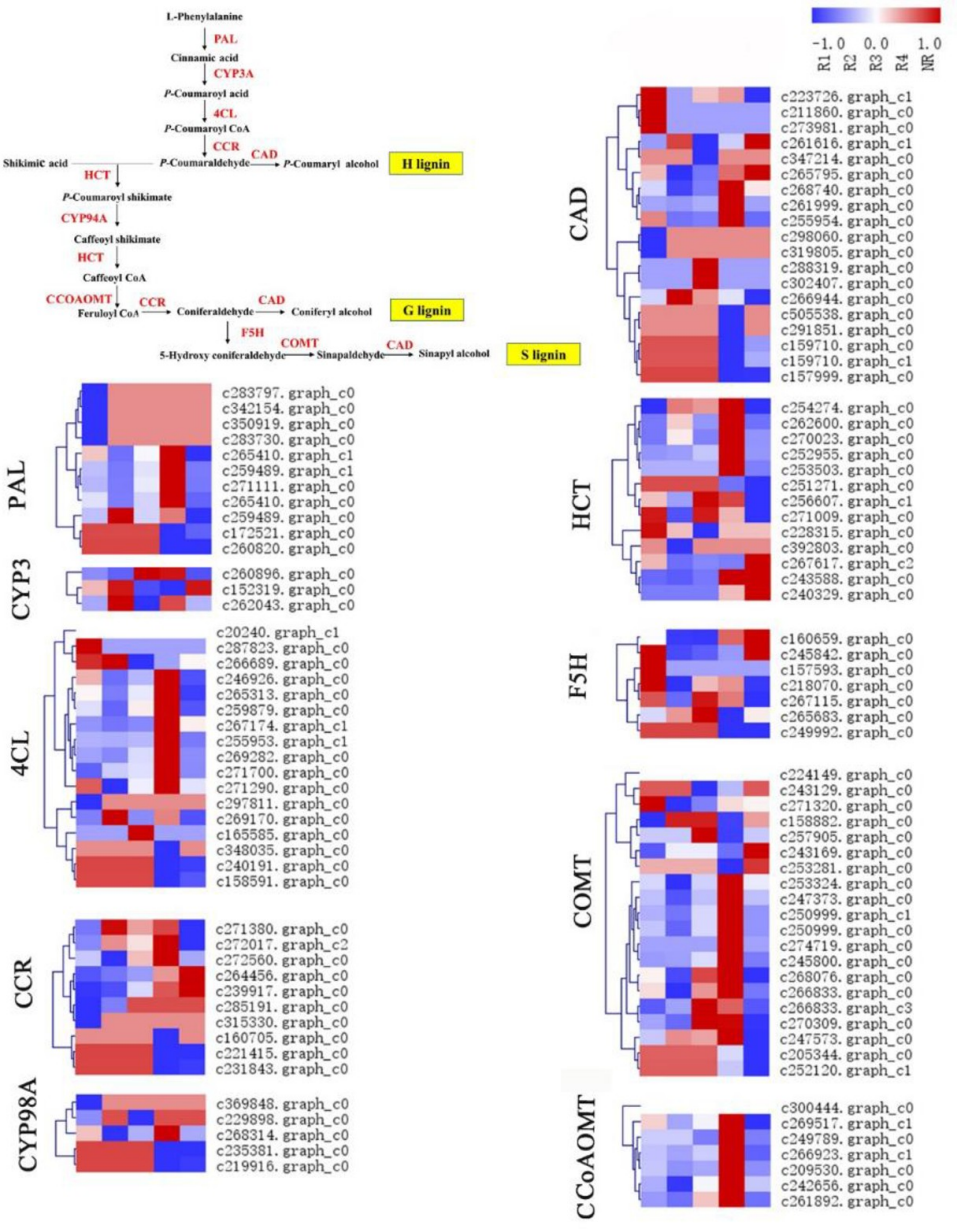
Heat map of phenylpropanoid biosynthesis-related genes in the five tissues.

Lignin plays a role in maintaining the structural integrity, strength, and hardness of the cell wall, aiding in the transport of water, prevention of cell wall permeation and protection of plants from pathogen infection. Lignin is mainly polymerized by three monomers, including coumaryl alcohol (H-lignin), coniferyl alcohol (G-lignin) and sinapyl alcohol (S-lignin). The sequencing results in the KEGG pathway database were annotated and analysed to obtain the key synthetase genes in the phenylpropane biosynthesis pathway (Fig 6). PAL initially directly catalyses the deamination of L-phenylalanine to produce cinnamic acid; CYP3A and 4CL catalyse hydroxylation and acetylation, respectively, and produce P-coumaroyl acid and P-coumaroyl CoA in turn. Under the action of HCT, CYP94A and F5H, P-coumaraldehyde can be converted to 5-hydroxyconiferaldehyde, which is important for the production of S-lignin. CCR and CAD produce cinnamaldehyde and cinnamyl alcohol, respectively, by catalytic reduction, and CAD can also produce P-coumaryl alcohol (as a precursor of H lignin). CCoAOMT and COMT catalyse the O-methylation reaction and eventually produce G-lignin and S-lignin. In addition, we also found that caffeic acid 3-O-methyltransferase (COMT) had the highest number of annotated genes, at 20, followed by cinnamyl-alcohol dehydrogenase (CAD), at 19; 4-coumarate—CoA ligase (4CL), at 17; shikimate O-hydroxycinnamoyltransferase (HCT), at 13; PAL, at 11; CCR, at 10; caffeoyl-CoA O-methyltransferase (CCoAOMT), at 7; ferulate-5-hydroxylase (F5H), at 7; 5-O-(4-coumaroyl)-D-quinate 3’-monooxygenase (CYP98A), at 5; and trans-cinnamate 4-monooxygenase (CYP3A), at 3.

Some PAL genes had the same variation trend. Nine genes were upregulated in R4, and four genes were significantly upregulated. Most genes are downregulated in R1. Most genes with CCR expression levels were higher in R4. For F5H, genes were mainly upregulated in R1 and R3. The expression levels of the COMT and CCoAOMT genes were similar; specifically, they were upregulated in R4, and downregulated in most other tissues.

### Validation of RNA-seq data by qRT-PCR

**Fig 7:**
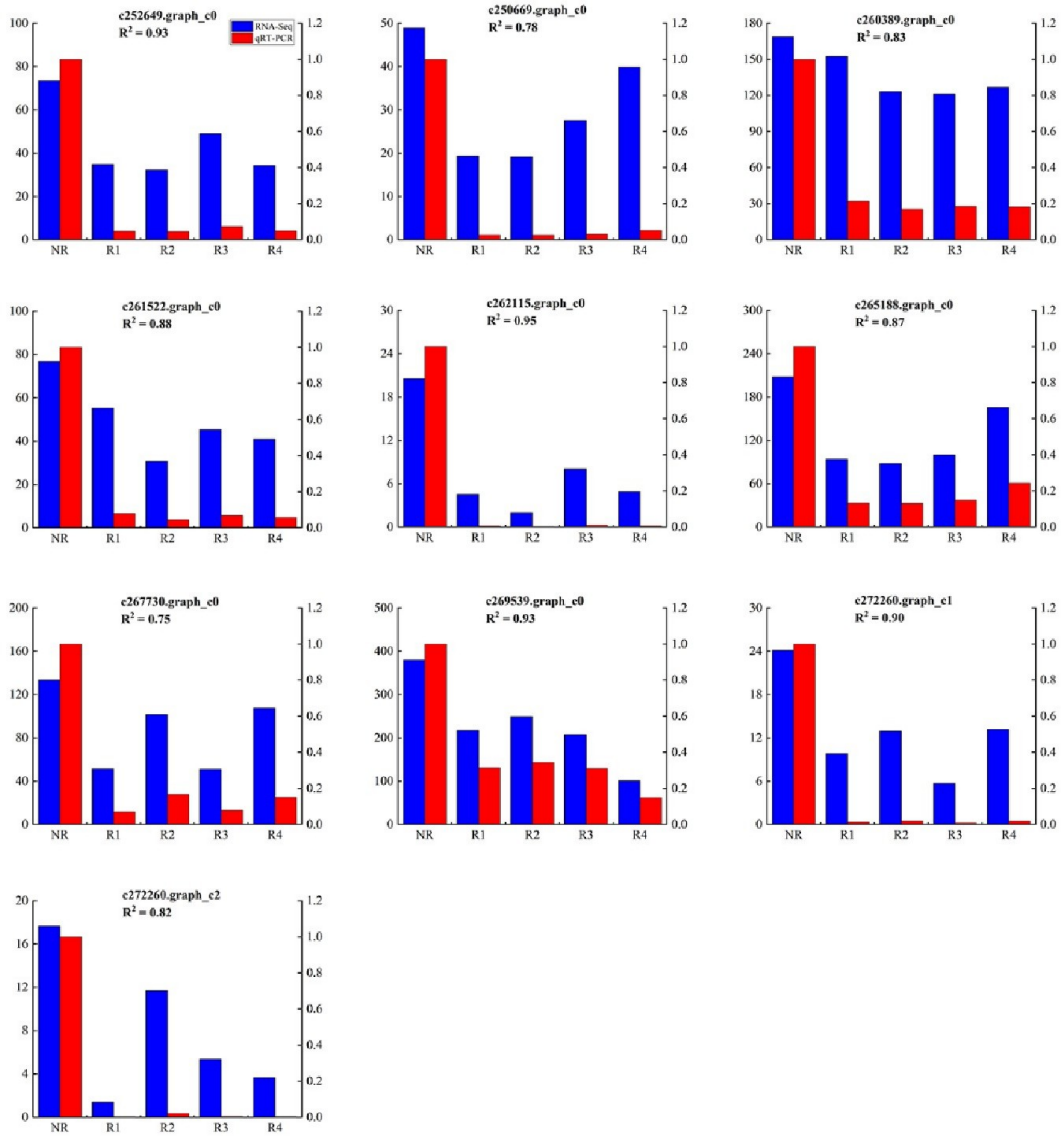
Comparison of qRT-PCR verification and FPKM value. The left y-axis indicates the FPKM value of ten differently expression genes in transcriptome. The right y-axis indicates the relative expression level of ten differently expression genes in qRT-PCR.

To verify the accuracy of the genes obtained by RNA-seq, we used c257504.graph_c0 as the internal reference gene and used the 2-ΔΔCT method to detect the expression levels of the 10 selected DEGs (Fig 7). qRT-PCR technology was used to verify that the expression levels of the R1, R2, R3, and R4 genes of Caucasian clover were consistent with the gene expression levels determined by RNA-seq. The trends of the two results are basically the same, demonstrating that the accuracy and validity of the RNA-Seq results are effective for data analysis.

## Discussion

Caucasian clover is a legume plant with special rhizomes, and previous studies have provided the full-length transcriptome of Caucasian clover rhizomes, revealing gene expression patterns and annotations in different tissues. We also explored the role of hormones, especially IAA, in rhizomes. However, there are few studies on the role of the phenylpropane biosynthesis pathway in different tissues of the Caucasian clover rhizome [29]. RNA-seq transcriptome data have accelerated our understanding of the complex system of gene transcription patterns, gene structural differences and gene regulatory networks. The data we obtained greatly enrich the transcriptome information of the Caucasian clover rhizome. Rhizomes are the absolute branches that initially form rhizome buds in uncertain positions of the lateral roots; then, they differentiate, grow and are finally removed from the matrix. This type of rhizome structure is evolutionarily formed via adaptation to natural conditions.

We used RNA-seq to obtain mapped reads of five tissues, where R1 reached a maximum of 17,497,291. R1 is the root node, which is particularly important for the formation of rhizomes. We divided the five tissues into two parts for DEG analysis. First, we compared the rootless tiller (NR) at the young seedling stage with the four tissues (R1, R2, R3 and R4) in the mature phase and then studied the DEGs in the four tissues (R1, R2, R3 and R4) in the mature phase.

Phenylpropanoids are very important for the entire process of rhizome development. Lignin widely exists in the secondary cell wall of vascular plants and can provide structural rigidity for plant uprightness, and its synthesis has been well studied in Melilotus albus [30]. Many key genes are involved in the lignin synthesis pathway, such as PAL, 4CL, CCR, CAD, HCT, F5H, COMT and CCoAOMT [31]. For example, CCR and CAD are key enzymes involved in the specific pathway of lignin synthesis [32, 33]. Previous studies have shown that CAD can change the structure of lignin but does not have much effect on the content of lignin [34]. As the first key enzyme in the biosynthetic pathway of lignin, PAL is the first rate-limiting enzyme in the phenylpropane biosynthesis pathway. It can catalyse the deamination of L-phenylalanine to generate cinnamic acid [35]. 4CL can acetylate P-coumaroyl acid to produce P-coumaroyl CoA ester, which is the last key enzyme in the phenylpropane biosynthesis pathway [36]. COMT and CCoAOMT are considered to have important regulatory effects on the synthesis of S-type lignin monomers and G-type lignin monomers, respectively [37, 47]. We identified 10 DEGs in the phenylpropane biosynthesis pathway. We found that most genes belonging to PAL, 4CL, COMT and CCoAOMT were upregulated in R4 compared with NR (Fig 7). The upregulation of the expression of these key genes involved in the phenylpropane biosynthesis pathway may lead to the promotion of lignin synthesis. These results may be related to the fact that R4 cells divide more actively than NR cells and constantly produce new cell walls.

During the young seedling stage of rootless tiller growth, starch and sucrose metabolism have important roles in early development, which may be to accumulate energy for subsequent growth and promote growth conditions. Genes related to plant hormone signal transduction are abundant in the mature phase after development is completed. These results are consistent with the results of studies on the development of lateral buds from the Phyllostachys edulis rhizome [38].

In the process of root development of Caucasian clover, the genes and pathways that regulate the development of each part are different. The high content of RAC1 in R1 has been confirmed to be related to cell proliferation [39]. In other plants, KAR1 plays an important role in the synthesis of fatty acids, especially type II fatty acids [40].

The IAA/ABA ratio can regulate praecox rhizome bud germination [40]. Peroxidase can determine the function of cell wall lignification, cell elongation, stress defence, phytohormonal regulation, desiccation and structural protein formation [41]. IAA, peroxidase, MYB and WRKY TF genes are highly expressed in R4, and these genes may be key bud growth genes or function in enhancing the resistance of buds to prepare for the growth of ground plants. Compared with the other three tissues at the mature phase, many genes in R4 were upregulated, for example, SAUR, GAUT, SUS and LOX (Fig 3). SAUR is an auxin response factor. The lack of SAUR function in Arabidopsis leads to shorter hypocotyls and decreased auxin transport [42, 43]. Therefore, we speculate that SAUR can positively regulate the growth of Caucasian clover root tip cells by regulating the transport of auxin. It has been reported that the GAUT gene family is of great significance for plant cell wall pectin biosynthesis, and we have also found high expression of the GAUT gene in Caucasian clover R4, which may have important significance for the synthesis of the Caucasian clover cell wall [44]. Another highly expressed gene in R4, LOX, was confirmed to increase the activity of LOX as the hypocotyl of sunflower seedlings elongated [45]. Therefore, we speculate that the upregulation of LOX in R4 may be related to the accumulation of lipids in the root tip. Previous research has shown that sucrose can control the upward bending of the red rice rhizome [46, 47]; however, we have identified highly expressed sucrose synthase (SUS) in R4 Caucasian clover, and we speculate that it may have significance in controlling the direction of rhizome development. Some studies have shown that MYB transcription factors regulate phenylpropane biosynthesis [48].

In NR, a highly expressed hemicellulose regulatory enzyme, GBE1 (1,4-alpha-glucan branching enzyme); MYB, which is related to secondary metabolism [49]; and 23 upregulated ethylene response factor (AP2/ERF) TFs that play essential regulatory roles in plant biotic and abiotic stress responses and secondary metabolism biosynthesis were upregulated [50]. However, the genes 4CL, CAD, COMT, and CCoAOMT, which are key enzymes in the lignin synthesis pathway, were mostly downregulated, which is normal for the initial stage of young roots [34]. Genes related to lignin synthesis were differentially expressed in different parts, but there was no obvious pattern. We speculate that some 4CL genes in R4 exhibited upregulated expression because isomers can guide metabolic flux through different pathways to synthesize various phenolic compounds, such as different monoethylene glycols, flavonoids and isoflavones [49]. CAD may change the structure of lignin without exerting much influence on the lignin content [34]. COMT has different effects on G lignin content and S lignin content in different plants, and its effects on lignin content specifically in Caucasian clover need to be determined [51].

## Conclusions

In summary, we reported the transcriptome of the mature phase of the Caucasian clover rhizome and annotated the transcripts. The expression levels in different tissues and annotation for the transcripts are provided. We analysed the specific expression of genes in different tissues and compared the differences between the NR and mature-phase rhizomes. In addition, we emphasized the role of the phenylpropane biosynthesis pathway in the rhizome. This study provides unique insights into the development of Caucasian clover, laying a molecular foundation for future research.

## Supporting information

S1 Fig All combination. Transcript length distribution.
(TIF)

S1 Table Primers used for qRT-PCR. (XLSX)

S2 Table Summary statistics of transcripts and unigenes. (XLSX)

S3 Table Output statistics of transcriptome sequencing. (XLSX)

S4 Table Statistics of differentially expressed genes. (XLSX)
