# Supplementary figures and images for: Transcriptome profiling unveils the mechanism of phenylpropane biosynthesis in rhizome development of Caucasian clover
Source: PLoS One. 2021 Jul 13;16(7):e0254669. doi: 10.1371/journal.pone.0254669 (PMC8277049; doi:10.1371/journal.pone.0254669)

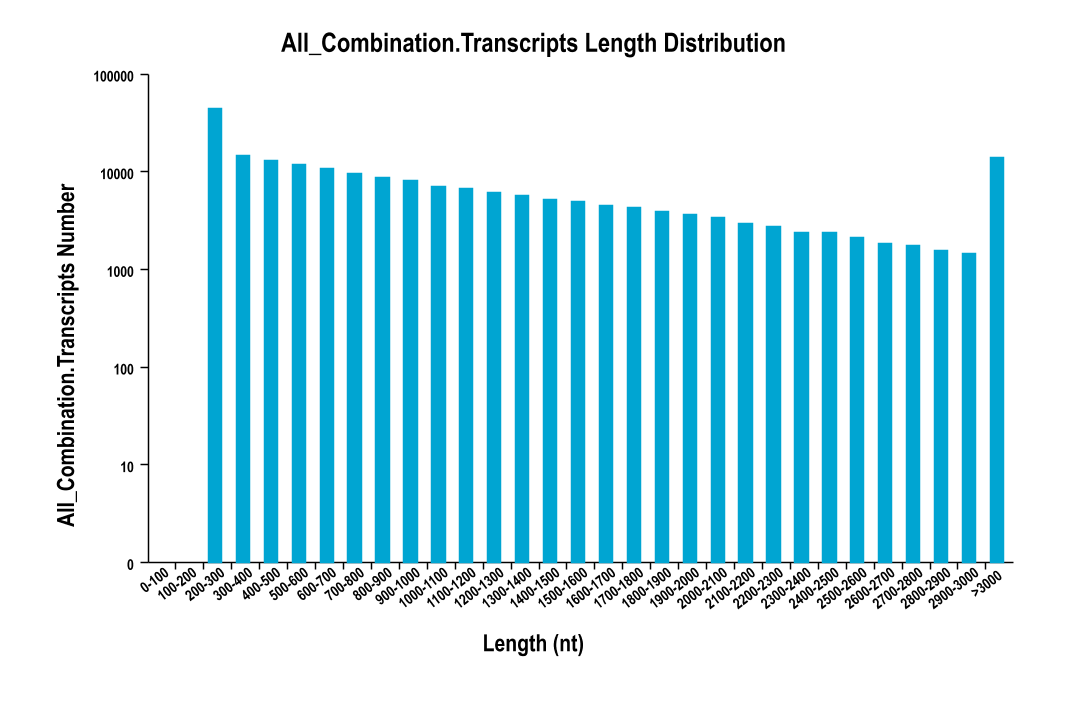

Supplement: S1 Fig — Transcript length distribution. (TIF) [file pone.0254669.s001.tif]
